# Silent Voices: Exploring Narratives of Women's Experiences of Health Care Professional Responses to Domestic Violence and Abuse

**DOI:** 10.1007/s10912-020-09621-x

**Published:** 2020-05-15

**Authors:** Julie McGarry, Kathryn Hinsliff-Smith

**Affiliations:** 1 School of Health Sciences, University of Nottingham, B Floor, South Block Link Queen’s Medical Centre, Nottingham, NG7 2HA UK; 2 De Montfort University, Leicster School of Nursing and Midwifery, Faculty of Health and Life Sciences, Edith Murphy Building Room 3.09, Leicetser, LE1 9BH UK

**Keywords:** Domestic violence and abuse, Narrative, Focus groups, Pedagogy, Healthcare professionals

## Abstract

The impact of domestic violence and abuse (DVA) is far reaching not least in terms of both the immediate and longer term physical and mental wellbeing of those who have experienced abuse. DVA also exerts a considerable detrimental impact on the wider family including children. While professional perspectives of working with DVA survivors is increasingly well documented, there remains a paucity of accounts of encounters with healthcare services and/or healthcare professionals from survivors of DVA themselves. A central aim of this study was the exploration of women’s experiences of healthcare encounters told purely as personal narrative rather than framed in more traditional research terms. The focus of this paper therefore is unedited personal stories of encounters with healthcare professionals. The position of narrative as research method and the presentation of narratives in this particular instance are also considered.

## Introduction

It is now well established that domestic violence and abuse (DVA) is a significant global societal and public health concern (World Health Organisation 2019). The impact of DVA is far reaching and impacts on both the immediate and longer term physical and mental wellbeing of those who have experienced abuse (McGarry and Nairn 2015; Trevillion et al. 2012; McGarry, Simpson, and Hinsliff-Smith 2011). DVA also exerts a considerable detrimental impact on the wider family including children (Hester, Pearson, and Harwin 2006).

There has been a growing awareness and acknowledgment of the scope and impact of DVA within the context of healthcare from the perspective of professionals across a range of settings including mental health (Dienemann et al. 2000), primary care (Feder et al. 2011) and the emergency department (McGarry and Nairn 2015). This has been further reinforced through national policy guidance, for example, in the UK context (National Institute of Health and Care Excellence (NICE) 2016). However, while professional perspectives of DVA are increasingly well documented, there remains a paucity of accounts of encounters with healthcare services and/or healthcare professionals from survivors of DVA themselves (Humphreys and Thiara 2003). This presents a significant deficit in current understanding, and one which this study has attempted to address.

### Why we chose narrative storytelling

The aim of this study was to explore survivors’ experiences of encounters with healthcare professionals as a direct consequence of DVA. Through the telling of personal stories or narrative, it was envisaged that these encounters, which historically have been largely invisible, would be made visible from the personal perspective of the women themselves. A central facet of this study was the exploration of women’s experiences of healthcare encounters told purely as personal narrative rather than framed in more traditional research terms such as, “How should healthcare professionals respond?” This approach is in keeping with Arthur Frank (2013) who, in *The Wounded Storyteller*, describes the essential value of ownership of life stories, albeit his work was situated within the context of physical ill health:

…taking the professional perspective undoes what *The Wounded Storyteller* is most concerned to bring about: a view from the ill person’s perspective, in which the central problem is how to avoid living a life that is diminished, whether by the disease itself or others responses to it. (16)

### Focus Groups

A focus group approach was facilitated with six women who identified themselves as survivors of DVA. We utilised this approach because it has been identified as supporting those who may not feel comfortable in a one-to-one interview (Wilkinson 1998), as facilitating a safe environment for discussion (Owen 2001), as supporting participants who may not feel they have anything of value to contribute (Kitzinger 1994, 1995), and as providing a social forum of interaction whereby the perspectives of the participants are dominant over the agenda of the researcher (Wilkinson 1998).

At the time of the study two of the women were residents within a refuge, and four women were living in their own homes without their partners or ex-partners. All of the women were receiving ongoing support from local DVA specialist services.

The appropriate research governance approvals were sought prior to the study taking place, as the focus group formed part of a larger service evaluation exploring support for survivors of DVA. Recruitment to the study was undertaken through the local DVA specialist services, and informed consent was sought prior to the focus group taking place. The focus group was undertaken in a mutually agreed location where support was available from specialist services for the women both during and following the focus group.

Prior to undertaking the focus group, we had approached the specialist DVA agency to gauge potential participants’ feelings towards the use of audio-recording equipment. The feedback that we received was that the women did not want the group meeting recorded, and so, as researchers (JM, KHS), we decided to take contemporaneous field notes. This approach has been used elsewhere and worked well in terms of capturing verbatim narrative and personal stories as they were shared within the group. We were also able to compare our notes at the end of the focus group for accuracy in capturing the discussions (Ziebland et al. 2013). We recognised the potential limitations of using this approach; however, we also felt that not attempting to give voice to the experiences of survivors only served to further compound their silence within the wider DVA discourse.

### Findings and reflections from the focus group

All of the women who attended the focus group were clearly motivated to take part, wanting to help professionals develop their practice and to support other women who had experienced DVA and who may come into contact with or access health services. For example, all of those who agreed to take part in the focus group described how they “wanted to help others” or considered “this might be useful [for healthcare professionals] in the future.” We were also mindful of the open forum nature of the focus group, and as such, while we had developed a broad topic guide, the shape and direction of the group discussions were participant led throughout.

In presenting the findings of the focus group, we have been mindful of the need to provide the forum through which the women, who are survivors of abuse, can narrate their own accounts of care encounters rather than the researchers editing these stories into a predominantly professional or researcher led dialogue. This approach echoes the work of Crawford et al. (2015) who clearly articulate the importance of drawing a distinction between “meaning” and “information” in seeking an understanding of an individual’s “life-world.” Essentially this means that an individual’s life experience is not readily reducible to a data-like format that is context neutral but is rather embodied and contextualised within a wider personal narrative or discourse. In so doing Crawford et al. highlight the centrality of meaning in the understanding of lived experience:

Matters are made meaningful when people understand and make sense of their actions, feelings and thoughts. Often this occurs through people creating narratives about themselves and events in their world. (4)

These sentiments and the onus on the pivotal role of meaning and context within narrative accounts have shaped the way in which we have chosen to present the focus group discussions.

In the following accounts, pseudonyms have been used to protect the anonymity of the participants.

### Jane’s story

In the focus group, the women spoke of the ways in which professionals and the perpetrator (or partner and, in one case, the wider family) had reinforced their experiences and perceptions of abuse as originating as their fault or as blameworthy themselves. Rani said, “It’s a family injury…in Asian families it doesn’t matter you just have to sit at home and cry, just your job.” Jane described how she “felt ashamed” and deemed the situation as “my fault.” She also spoke of how the perpetrator (her partner) had used her prior mental health problems, depression and anxiety, as a way, in her words, of “controlling” her actions or “threatening her.” For example, he told her, “I’ll tell them that you’re mental again.” This compounded Jane’s own feelings in that “if you have a mental illness then it is less likely for people to believe you.” Jane spoke about her home life within the context of her early pregnancy, which she described as not planned as “it was not when I would have chosen to have a baby.” It was during this time that her partner told her he would tell everyone that she was “mental and no one would believe her if she told them about [the abusive nature of their relationship].” Jane’s greatest fear was that her baby would be taken away from her, and so she did not feel able to tell any of the professionals about the abuse. Another participant, Anne, also told the group how she was scared to tell healthcare professionals about her abusive partner because she was afraid of social services “taking away her children.”

Throughout the focus group, it was clear that the women blamed themselves for the circumstances they found themselves in and their “irrational behaviour” at home. This was often reinforced and compounded by the perpetrator. However, the women in the group also spoke of this sense of blame sometimes, perhaps unwittingly, reinforced by professionals. For example, Jane spoke of how, after speaking to her partner, healthcare professionals became concerned that her pregnancy may affect her mental health status and that her midwife referred her to a specialist mental health unit at the local hospital. She experienced anxiety that “she [the midwife] wrote in her notes [about previous mental health issues]” and that “my husband was aware and got me into trouble.” Jane further explained that she felt that her husband got her into “trouble,” both by telling the midwife about her previous mental health history and “because I became ill [from increased anxiety].” Jane also told the group that when she told her psychiatrist during this period of care that her husband wouldn’t help with household chores, he suggested [jokingly] “using sex for favours like doing chores” [Jane’s partner had used sexual violence in their relationship previously].

### Sheila’s story

For Sheila, the severity of her injuries were such that the emergency department staff at her local hospital “pleaded” with her not to return home and how she was provided with support and “advice.” However, as she had attended the emergency department “many times,” she thought she was “wasting their time,” but “nobody ever said that outright.” Sheila had a mixed impression from staff in that they were both “lovely to me” but also that “some nurses [were] not sympathetic, well that’s how I felt.” Sheila said she felt guilty that she couldn’t take their advice and leave.

In the focus group, notions of ‘time wasting’ were bound up with perceptions of the ‘hiddenness’ of their abuse which several of the women talked about, albeit in different ways. Maria told the group how her ex-partner “ran over her” in the car, causing a serious knee injury. The next day Maria attended a local emergency department (ED) and was asked repeatedly by the staff how the injury had occurred. Maria chose not to disclose that her husband had run her over with his car, and when asked responded “I don’t know” and “I told them I hurt it on the stairs.” It was clear Maria said, that the staff were perplexed by the nature of the injury and found it difficult to understand how this could have happened as a result of an accidental injury. Maria felt a tremendous sense of guilt as ED staff sent her for “all kinds of tests,” and “I could have stopped it all by telling the truth…but couldn’t…the doctors said I could leave but I felt guilty for [the] unnecessary tests.”

### Group dialogue

During the focus group discussions, women spoke of how they had accessed health services but that they hadn’t always felt able to disclose their abuse. Rani had attended the ED on the first occasion due to her feeling that her health was deteriorating, and “[I was] depressed with [my] husband.” “It was affecting me emotionally, physically, and he had financial control.” She didn’t tell ED staff and left with advice to arrange to see her general practitioner. Gita said that even if she had been asked, “it’s difficult to come out with the words.” Jane spoke of a similar experience, of not being able to say outright that she was experiencing abuse and that she had said to staff, “I kept saying, but I need more support,” and after a serious post-birth trauma had been advised that she needed to rest. She told staff “I can’t rest in bed, he won’t help and I need more support.” “How could they not think that something was wrong then?”

All of the women in the focus group had been recipients of healthcare as a result of DVA. Most of the women had attended the ED at one time or another. The ED environment was “open, [there was] no privacy, felt rushed”; “The staff were always very busy.” It was also apparent that women chose to remain hidden within services for fear of retaliation from the perpetrator. While Sheila describes herself as “a repeat attender,” she was unaware of the existence of DVA support services as she always attended ED alone and at night to avoid recognition. While Anne was scared to keep attending ED as she would “get a good hiding” when returning home and questioned about what she said to staff by her partner. Injuries were also hidden, with Jane stating that “with sexual violence you can hide it.” Anne returned home, despite her injuries because of her dogs. Her father suspected but she never told or spoke to family about the abuse, “so [I] had to hide it.” Gita was given [helpline] cards by healthcare professionals [about services] but “was scared to call” and never did, but her husband found them and asked “why have you got them?”

The responses to disclosure that women described in the focus group highlighted the ways in which they explained how they were placed in a largely impossible situation of responsibility and “frustration” on the part of the professionals that a woman did not remove herself from the situation. This is linked in part to the earlier themes and the sense of ‘time wasting.’ However, women also spoke of the practical difficulties of leaving with Rani describing how she was unable to use her mobile phone as it was registered to her husband and that “husband had control…was living in a jail.”

Women spoke of the ways in which professionals had tried to help them. There are clearly elements of frustration on the part of the professionals as Anne describes being told “you need to take control.” Professionals also appeared in some instances to be at a loss as what to do with when faced with a disclosure of DVA. For example, Anne described an attempted strangulation and how she “spoke to my GP who referred me to A&E because of concern about any physical damage to my neck” and “made suggestions but nothing more.” The lack of effective professional intervention or support following disclosure was highlighted by another focus group participant when she experienced blunt professional opinion in the sense of “you will be dead next time.”

However, within the focus group discussion, women also spoke of how healthcare professionals had taken a more pro-active stance; for example where the focus group participant described how the female psychiatrist and female general practitioner made me “strong” to leave. For Sheila when she visited her general practitioner on the second occasion, she disclosed she was experiencing DVA. When she attended again for a third time with a scar on her face, he “helped me to ring the Women’s Centre.”

### Concluding thoughts and reflections

In this study we have attempted to explore the experiences of women as survivors of abuse within the context of healthcare encounters through the use of narrative. We have aimed to present the findings of the study as unedited personal stories rather than formalised researcher accounts told from the perspective of women survivors of DVA themselves. As Bassett and Stickley (2010) describe, we believe ‘that the stories should speak for themselves’ (5). However, in so doing we are also aware of the potential methodological controversy that surrounds this approach.

There are a number of interpretive lenses through which a researcher may view the process of empirical enquiry, and these have frequently been defined as entailing either a qualitative or quantitative approach. Historically, these approaches have often been conceptualised as occupying divergent paradigms; for example, positivism *versus* naturalism, realist *versus* relativist, qualitative *versus* quantitative (Avis 1995). However, while individual beliefs regarding the nature of knowledge and how knowledge can be uncovered undoubtedly shape the way the researcher views the world, it may also be asserted that the preoccupation with notions of incommensurable research paradigms (Kuhn 1970) may jeopardise the quality of research, in terms of stifling dialogue regarding issues relating to the appropriateness of the chosen method for the field of enquiry. Although recognised as diverse in nature (Grbich 1999), qualitative approaches to research enquiry have been described as encompassing an interpretive and naturalistic approach (Denzin and Lincoln, 2000), underpinned by a philosophy that places the emphasis on the meanings that individuals attach to their social world (Bowling 1997).

Recently however, within the specific context of qualitative methodology, the debate has shifted from one which largely focuses on the concept of opposing paradigms to one which centres on claims to knowledge and notions of validity. The use of narrative or storytelling is one such approach which occupies a contested space with the main focus of criticism resting largely on notions of validity (Polkinghorne 2007). However, it has been argued that validity within the context of narrative texts occupies a particular position, as Lieblich, Tuval-Mashiach, and Zilber (1998) describe:

We do not advocate total relativism that treats all narratives as texts of fiction…We believe that stories are constructed around a core of facts or life events, yet allow a wide periphery for freedom…selection, addition to, emphasis on and interpretation of these “remembered facts.” (8)

As Polkinghorne further highlights, while there are potential threats to validity across all areas of research, there may be threats which are particular to narrative accounts, for example, the inadequacy of words to convey the nuances of personal experience or ‘the limits of reflection to bring notice to the layers of meaning that are present outside of awareness’ (2007, 480).

However, despite the potential limitations and contested space occupied by narrative and storytelling within qualitative methods, it is also clear that it is increasingly being recognised as a valuable and powerful asset for healthcare education and practice. This is, in part, due to the recognition that stories cannot be de-contextualised and that their core value lies in the ability to help situate professionals within the experience itself: “The potential and value of story lies not only in its use as a tool for reflection…but in its ability to represent and organise experience (Edwards 2014, 2).

As previously identified, women who have experienced DVA may present to a number of health services either as the direct result of an injury or through associated trauma—for example, mental ill-health. To date, however, while there is a growing professionally-driven evidence base surrounding healthcare encounters with survivors of DVA, the voices of survivors themselves remains largely unheard. The narratives presented in this paper illustrate the experiences of women as survivors within the healthcare system where their overriding sense of engagement was one of ‘timewasting’ and of 'guilt' at not being able to comply with professional advice. There was also a sense of invisibility within healthcare encounters; this was partially aligned to notions of survival with the perpetrator on the part of the woman but was also based in part on healthcare professionals failure to recognise women’s presentations in terms of potentially signalling DVA.

Taken as a whole, the narratives presented here also perhaps tellingly highlight that healthcare services and systems in many instances operate on the basis of a strong individual agency. Within this context the onus is placed on the individual within a particular system to both articulate their needs and to take action for example, as highlighted in the present study, responsibility to ‘take control.’ However, this paper also demonstrates that for DVA survivors in their encounters within healthcare this is not always possible and more importantly this study illuminates why this might be the case.

In conclusion, as the gaze of healthcare policy and practice becomes more focused towards supporting survivors of DVA it is crucial that healthcare professionals are able to recognise the organisational and the less tangible barriers that face survivors of DVA within the health system. It is suggested that through exploring the narratives of women as survivors of DVA as a starting point, healthcare professionals can begin to reflect on how those barriers may be overcome in the future.
